# Histamine H_1_ Receptor-Mediated JNK Phosphorylation Is Regulated by G_q_ Protein-Dependent but Arrestin-Independent Pathways

**DOI:** 10.3390/ijms25063395

**Published:** 2024-03-17

**Authors:** Shotaro Michinaga, Ayaka Nagata, Ryosuke Ogami, Yasuhiro Ogawa, Shigeru Hishinuma

**Affiliations:** Department of Pharmacodynamics, Meiji Pharmaceutical University, 2-522-1 Noshio, Kiyose, Tokyo 204-8588, Japan

**Keywords:** β-arrestin2, c-Jun N-terminal kinase, G_q_ protein, histamine H_1_ receptor, mitogen-activated protein kinase, protein kinase C

## Abstract

Arrestins are known to be involved not only in the desensitization and internalization of G protein-coupled receptors but also in the G protein-independent activation of mitogen-activated protein (MAP) kinases, such as extracellular signal-regulated kinase (ERK) and c-Jun N-terminal kinase (JNK), to regulate cell proliferation and inflammation. Our previous study revealed that the histamine H_1_ receptor-mediated activation of ERK is dually regulated by G_q_ proteins and arrestins. In this study, we investigated the roles of G_q_ proteins and arrestins in the H_1_ receptor-mediated activation of JNK in Chinese hamster ovary (CHO) cells expressing wild-type (WT) human H_1_ receptors, the G_q_ protein-biased mutant S487TR, and the arrestin-biased mutant S487A. In these mutants, the Ser487 residue in the C-terminus region of the WT was truncated (S487TR) or mutated to alanine (S487A). Histamine significantly stimulated JNK phosphorylation in CHO cells expressing WT and S487TR but not S487A. Histamine-induced JNK phosphorylation in CHO cells expressing WT and S487TR was suppressed by inhibitors against H_1_ receptors (ketotifen and diphenhydramine), G_q_ proteins (YM-254890), and protein kinase C (PKC) (GF109203X) as well as an intracellular Ca^2+^ chelator (BAPTA-AM) but not by inhibitors against G protein-coupled receptor kinases (GRK2/3) (cmpd101), β-arrestin2 (β-arrestin2 siRNA), and clathrin (hypertonic sucrose). These results suggest that the H_1_ receptor-mediated phosphorylation of JNK is regulated by G_q_-protein/Ca^2+^/PKC-dependent but GRK/arrestin/clathrin-independent pathways.

## 1. Introduction

G protein-coupled receptors (GPCRs) comprise the largest family of plasma membrane receptors and play crucial roles in regulating cellular responses to physiological ligands and drugs [1,2,3]. Arrestins are known to be involved not only the desensitization and internalization of GPCRs but also in G protein-independent signal transduction [4,5,6,7,8,9,10]. Briefly, active forms of GPCRs are phosphorylated by G protein-coupled receptor kinases (GRKs), followed by the binding of arrestins to phosphorylated receptors to uncouple the receptors from G proteins. Arrestins mediate the internalization or sequestration of receptors from the cell surface via the formation of clathrin-coated pits, followed by endocytic vesicle scission via dynamin, a mechanochemical GTPase. Arrestins also act as scaffolding proteins to activate mitogen-activated protein (MAP) kinases, such as p42/p44 extracellular signal-regulated kinases (ERK), c-Jun N-terminal kinase (JNK)/stress-activated protein kinase (SAPK), and p38, to mediate various cellular responses, including proliferation and inflammation. Agonists that preferentially activate signal transduction pathways via G proteins or arrestins are known as biased agonists [11,12,13].

G_q_ protein-coupled histamine H_1_ receptors are known to mediate various physiological and pathophysiological responses, such as allergy and inflammation in peripheral tissues and arousal and memory in the central nervous system [14,15,16,17,18,19,20]. Although H_1_ receptors appear to mediate the activation of MAP kinases [21,22,23,24,25,26,27,28,29,30,31,32,33,34,35,36,37], it is not well understood how the H_1_ receptor-mediated activation of MAP kinases might be differentially regulated by G_q_ proteins and arrestins.

During the course of our investigation on mechanisms of the desensitization and internalization of H_1_ receptors [38,39,40,41,42,43], we identified a G_q_ protein-biased human H_1_ receptor mutant S487TR, in which the Ser487 residue at the end of the C-terminus was truncated, and an arrestin-biased mutant S487A, in which Ser487 was mutated to alanine [42]. Histamine stimulated phosphatidylinositol turnover in Chinese hamster ovary (CHO) cells expressing S487TR but not S487A, while histamine stimulated the clathrin-mediated internalization of S487A but not S487TR. Using CHO cells expressing these H_1_ receptor mutants, we found that histamine-induced ERK phosphorylation was differentially regulated by G_q_ proteins and arrestins, i.e., G_q_ protein/Ca^2+^/protein kinase C (PKC)- and GRK/arrestin/clathrin-mediated pathways, respectively [43]. However, it is unclear whether the H_1_ receptor-mediated phosphorylation of JNK is also regulated by G_q_ proteins and arrestins in a manner similar to ERK phosphorylation. Here, we present our findings that the H_1_ receptor-mediated phosphorylation of JNK is regulated by G_q_ protein/Ca^2+^/PKC-dependent but GRK/arrestin/clathrin-independent pathways.

## 2. Results

### 2.1. Histamine Induces JNK Phosphorylation in CHO Cells Expressing S487TR but Not S487A

**Figure 1:**
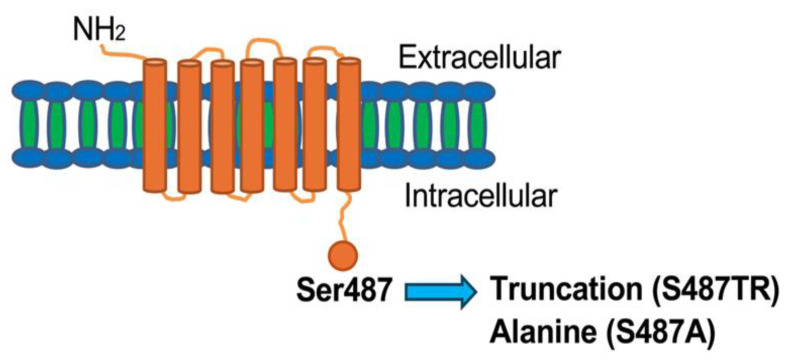
Schematic diagram of the C-terminal mutants of human histamine H_1_ receptor used in this study. Chinese hamster ovary (CHO) cells expressing wild-type (WT) human histamine H_1_ receptors tagged with three molecules of hemagglutinin at the N-terminal and its C-terminal mutants, S487TR and S487A. In the mutants, the Ser487 residue located at the end of the intracellular C-terminal of the WT receptor was truncated or mutated to alanine, respectively. S487TR and S487A appeared to be G_q_ protein- and arrestin-biased H_1_ receptors, respectively.

**Figure 2:**
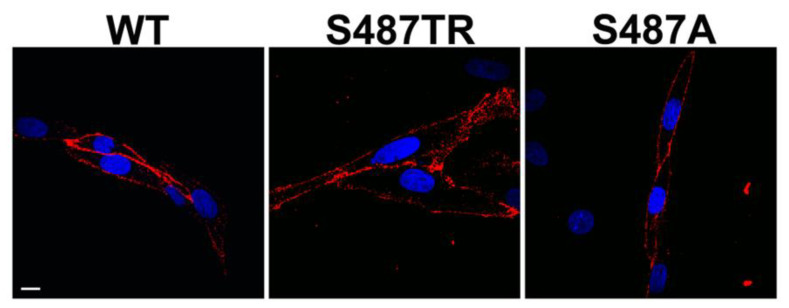
Confocal immunofluorescence microscopy to detect WT, S487TR, and S487A H_1_ receptors expressed in CHO cells. Red fluorescence shows WT, S487TR, and S487A H_1_ receptors labeled with anti-hemagglutinin (HA) antibodies. Blue fluorescence shows nuclei labeled with 4′,6-diamidino-2-phenylindole (DAPI). Scale bar 10 μm.

Figure 1 shows a schematic diagram of the C-terminal mutants of human histamine H_1_ receptors used in this study, in which S487TR and S487A appear to represent the G_q_ protein- and arrestin-biased mutants, respectively [42,43]. Confocal immunofluorescence microscopy showed that wild-type (WT), S487TR, and S487A H_1_ receptors were predominantly expressed on the cell surface of CHO cells (Figure 2). There were no significant differences between the total and phosphorylated JNK levels in CHO cells with or without H_1_ receptor expression, but histamine-stimulated JNK phosphorylation was only observed in CHO cells expressing H_1_ receptors (Supplemental Figure S1).

**Figure 3:**
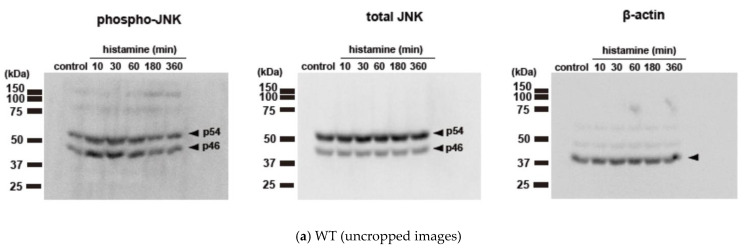

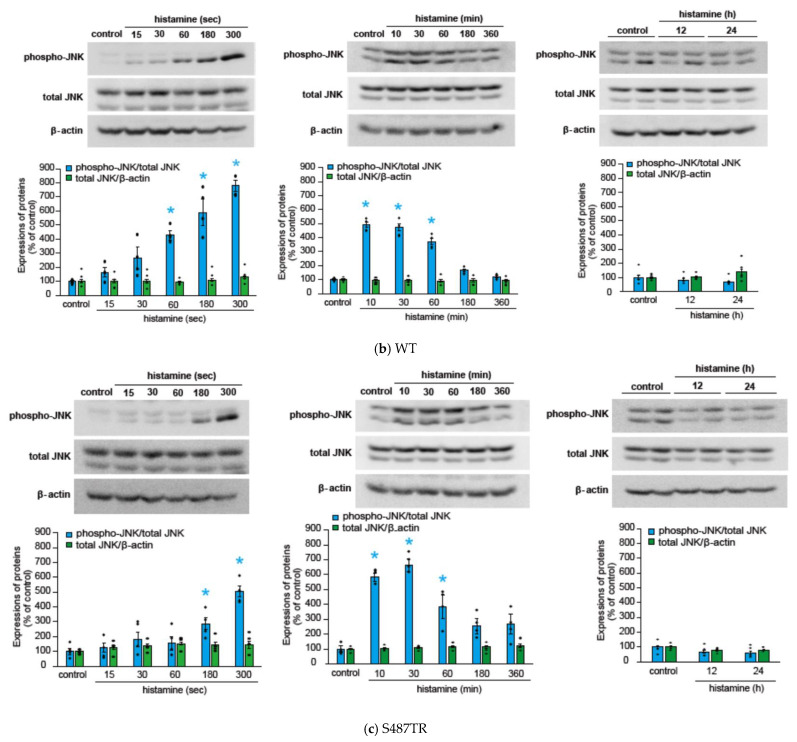

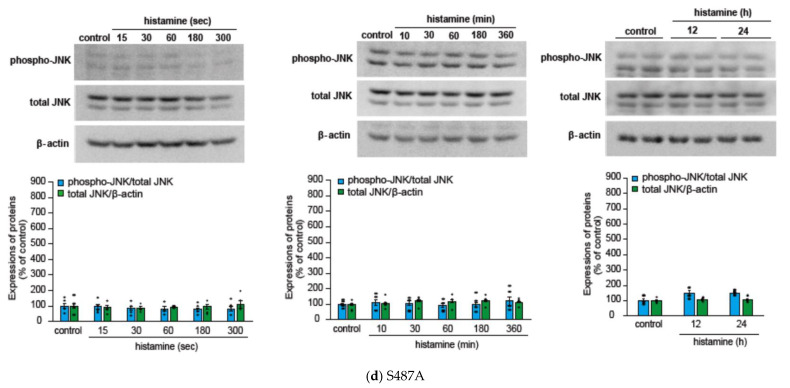
Time courses of histamine-induced JNK phosphorylation. (**a**) CHO cells expressing WT were stimulated with or without (control) 100 µM histamine for 10–360 min, and then protein extracts from the cells were subjected to immunoblot analyses. Typical uncropped immunoblot images of phosphorylated JNK (phospho-JNK) (**left**), total JNK (**middle**), and β-actin (**right**) are shown. Arrowheads indicate target proteins (phospho-JNK and total JNK, p46 and p54; β-actin, 40 kDa). The molecular weights are shown on the left side of images. (**b**–**d**) CHO cells expressing WT (**b**), S487TR (**c**), and S487A (**d**) were stimulated with or without (control) 100 µM histamine for 15–300 sec (**left**), 10–360 min (**middle**), and 12–24 h (**right**), and then protein extracts from the cells were subjected to immunoblot analyses. Typical immunoblot images of phosphorylated JNK (phospho-JNK), total JNK, and β-actin are shown in the upper panels in (**b**–**d**). Histamine-induced changes in the ratios of phosphorylated JNK to total JNK (phospho-JNK/total JNK) and total JNK to β-actin (total JNK/β-actin) are shown as percentages of the control in the lower graphs in (**b**–**d**). Assays were repeated four times. Values represent mean ± SE of data obtained from four independent protein samples. Individual data are shown as scatter plots. * *p* < 0.05 vs. control; one-way ANOVA and Dunnett’s test.

We initially evaluated the time course of histamine-induced JNK phosphorylation in CHO cells expressing WT, S487TR, and S487A (Figure 3), because our previous study suggested that G_q_ proteins and arrestins regulate the early and late phases of histamine-induced ERK phosphorylation, respectively [43]. In CHO cells expressing the WT receptors (Figure 3a,b), histamine-induced JNK phosphorylation occurred rapidly within 1 min and was maintained for 60 min. In CHO cells expressing S487TR (Figure 3c), histamine-induced JNK phosphorylation occurred rapidly within 3 min and was maintained for 60 min. In contrast, in CHO cells expressing S487A (Figure 3d), histamine failed to induce significant JNK phosphorylation during the period examined (up to 24 h). Thus, JNK phosphorylation was induced by histamine in CHO cells expressing WT and S487TR but not in those expressing S487A. These results suggest that histamine-induced JNK phosphorylation is mediated by G_q_ proteins, but not by arrestins, in CHO cells. It is possible that the relatively slower and lower level of phosphorylated JNK in CHO cells expressing S487TR compared to WT might be due to the lower expression level of S487TR compared to WT in these CHO cells, since our previous study indicated that the expression levels of WT, S487TR, and S487A in these CHO cells were approximately 300, 150, and 400 fmol/mg whole cell protein, respectively [42]. It is noted that p46 phosphorylation appeared to precede p54 phosphorylation in response to histamine (Supplemental Figure S2). Uncropped immunoblot images obtained from four independent experiments are shown in Supplemental Figure S3.

In subsequent experiments, we evaluated the effects of various inhibitors on JNK phosphorylation induced by histamine treatment for 30 min in CHO cells expressing WT or S487TR.

### 2.2. Histamine-Induced JNK Phosphorylation Is Mediated by G_q_ Protein/Ca^2+^/PKC-Dependent but GRK/Arrestin/Clathrin/Dynamin-Independent Pathway via Activation of H_1_ Receptors in CHO Cells Expressing WT and S487TR

**Figure 4:**
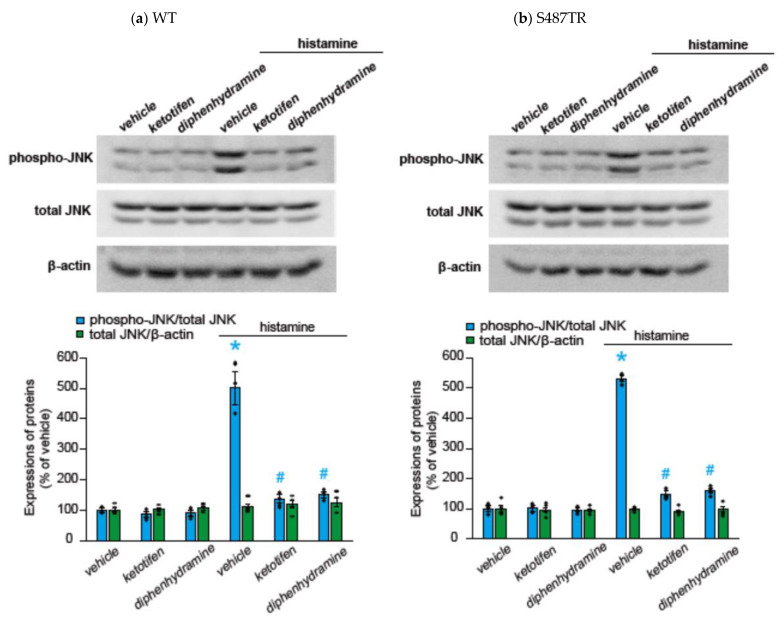
Effects of histamine H_1_ receptor antagonists on histamine-induced JNK phosphorylation. CHO cells expressing WT (**a**) and S487TR (**b**) were stimulated with or without 100 µM histamine for 30 min in the presence and absence (vehicle) of histamine H_1_ receptor antagonists, ketotifen (1 mM) or diphenhydramine (1 mM). Subsequently, protein extracts from the cells were subjected to immunoblot analyses. Typical immunoblot images of phosphorylated JNK (phospho-JNK), total JNK, and β-actin are shown in the upper panels in (**a**,**b**). Histamine-induced changes in the ratios of phosphorylated JNK to total JNK (phospho-JNK/total JNK) and total JNK to β-actin (total JNK/β-actin) are shown as percentages of the control (vehicle) without histamine treatment in the lower graphs in (**a**,**b**). Assays were repeated four times. Values represent mean ± SE of data obtained from four independent protein samples. Individual data are shown as scatter plots. * *p* < 0.05 vs. vehicle, ^#^ *p* < 0.001 vs. histamine (vehicle); one-way ANOVA and Tukey’s test.

To explore whether histamine-induced JNK phosphorylation was mediated by H_1_ receptors, we examined the effects of H_1_ receptor antagonists (antihistamines) on histamine-induced JNK phosphorylation in CHO cells expressing WT or S487TR (Figure 4). Ketotifen (1 mM) and diphenhydramine (1 mM) completely inhibited histamine-induced JNK phosphorylation in CHO cells expressing the WT (Figure 4a) and S487TR (Figure 4b). Thus, we confirmed that histamine-induced JNK phosphorylation was mediated by the activation of H_1_ receptors expressed in CHO cells.

**Figure 5:**
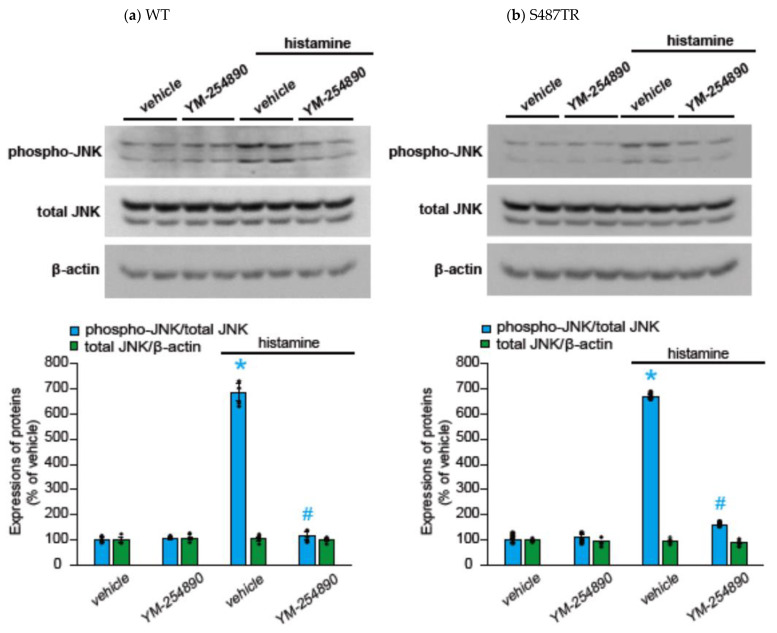
Effects of G_q_ protein inhibitor on histamine-induced JNK phosphorylation. CHO cells expressing WT (**a**) and S487TR (**b**) were stimulated with or without 100 µM histamine for 30 min in the presence and absence (vehicle) of a G_q_ protein inhibitor, YM-254890 (20 µM). Subsequently, protein extracts from the cells were subjected to immunoblot analyses. Typical immunoblot images of phosphorylated JNK (phospho-JNK), total JNK, and β-actin are shown in the upper panels in (**a**,**b**). Histamine-induced changes in the ratios of phosphorylated JNK to total JNK (phospho-JNK/total JNK) and total JNK to β-actin (total JNK/β-actin) are shown as percentages of the control (vehicle) without histamine treatment in the lower graphs in (**a**,**b**). Assays were repeated twice. Values represent mean ± SE of data obtained from four independent protein samples. Individual data are shown as scatter plots. * *p* < 0.05 vs. vehicle, ^#^ *p* < 0.05, vs. histamine (vehicle); one-way ANOVA and Tukey’s test.

**Figure 6:**
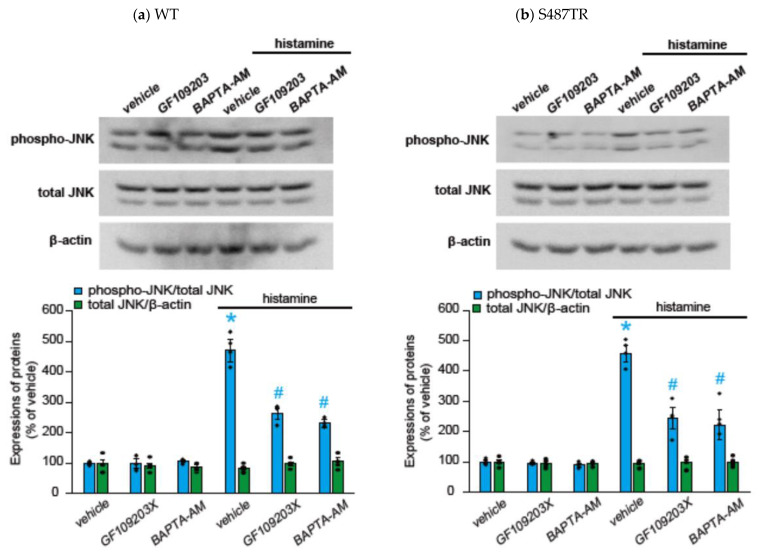
Effects of intracellular Ca^2+^ chelator and PKC inhibitor on histamine-induced JNK phosphorylation. CHO cells expressing WT (**a**) and S487TR (**b**) were stimulated with or without 100 µM histamine for 30 min in the presence and absence (vehicle) of an intracellular Ca^2+^ chelator (BAPTA-AM; 50 µM) or a PKC inhibitor (GF109203X; 10 µM), and then protein extracts from the cells were subjected to immunoblot analyses. Typical immunoblot images of phosphorylated JNK (phospho-JNK), total JNK, and β-actin are shown in the upper panels in (**a**,**b**). Histamine-induced changes in the ratios of phosphorylated JNK to total JNK (phospho-JNK/total JNK) and total JNK to β-actin (total JNK/β-actin) are shown as percentages of the control (vehicle) without histamine treatment in the lower graphs in (**a**,**b**). Assays were repeated four times. Values represent mean ± SE of data obtained from four independent protein samples. Individual data are shown as scatter plots. * *p* < 0.05 vs. vehicle, ^#^ *p* < 0.05 vs. histamine (vehicle); one-way ANOVA and Tukey’s test.

To explore whether H_1_ receptor-mediated JNK phosphorylation might involve G_q_ protein-mediated signal transduction processes, we examined the effects of a G_q_ protein inhibitor (20 µM YM-254890), an intracellular Ca^2+^ chelator (50 µM BAPTA-AM), and a PKC inhibitor (10 µM GF109203X) on histamine-induced JNK phosphorylation in CHO cells expressing WT and S487TR (Figure 5 and Figure 6). These inhibitors significantly suppressed histamine-induced JNK phosphorylation in these CHO cells. These results suggest that histamine-induced JNK phosphorylation is mediated by a G_q_ protein/Ca^2+^/PKC-dependent pathway.

**Figure 7:**
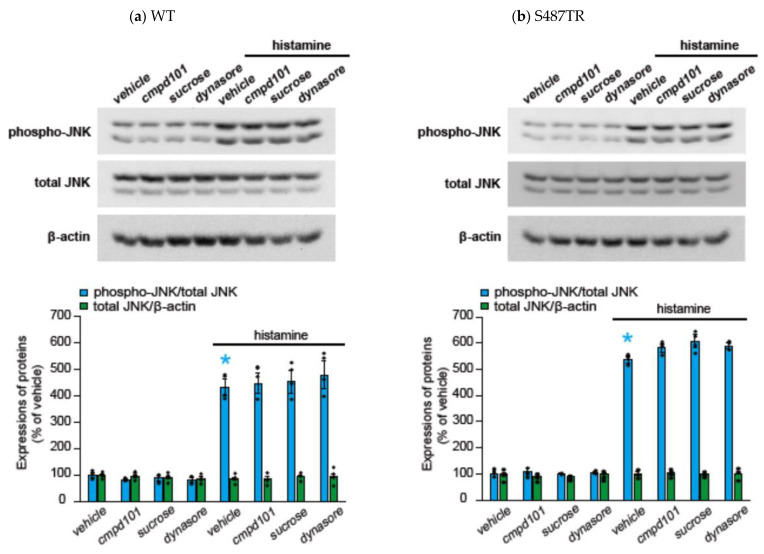
Effects of inhibitors against GRK, clathrin, and dynamin on histamine-induced JNK phosphorylation. CHO cells expressing WT (**a**) and S487TR (**b**) were stimulated with or without 100 µM histamine for 30 min in the presence and absence (vehicle) of an inhibitor of GRK2/3 (cmpd101; 30 µM), clathrin (a high concentration of sucrose; 0.32 M), or dynamin (dynasore; 100 µM). Subsequently, these protein extracts from the cells were subjected to immunoblot analyses. Typical immunoblot images of phosphorylated JNK (phospho-JNK), total JNK, and β-actin are shown in the upper panels in (**a**,**b**). Histamine-induced changes in the ratios of phosphorylated JNK to total JNK (phospho-JNK/total JNK) and total JNK to β-actin (total JNK/β-actin) are shown as percentages of the control (vehicle) without histamine treatment in the lower graphs in (**a**,**b**). Assays were repeated four times. Values represent mean ± SE of data obtained from four independent protein samples. Individual data are shown as scatter plots. * *p* < 0.05 vs. vehicle; one-way ANOVA and Tukey’s test.

**Figure 8:**
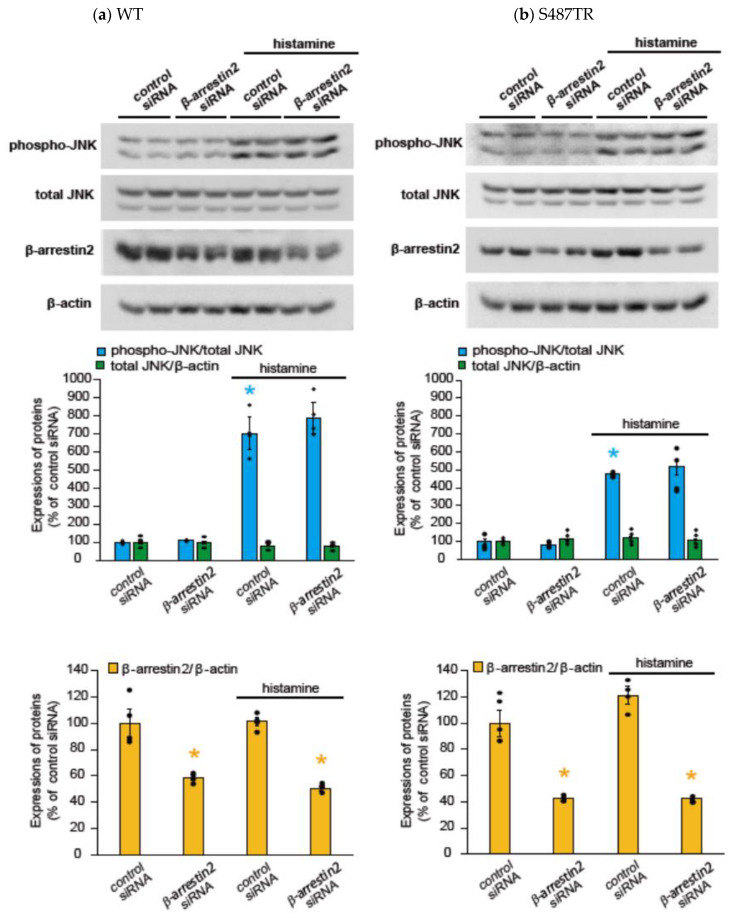
Effects of β-arrestin2 knockdown on histamine-induced JNK phosphorylation. CHO cells expressing WT (**a**) and S487TR (**b**) were treated with control siRNA or β-arrestin2 siRNA for 48 h and then stimulated with or without 100 µM histamine for 30 min. Protein extracts from the cells were then subjected to immunoblot analyses. Typical immunoblot images of phosphorylated JNK (phospho-JNK), total JNK, β-arrestin2, and β-actin are shown in the upper panels in (**a**,**b**). Histamine-induced changes in the ratios of phosphorylated JNK to total JNK (phospho-JNK/total JNK) and total JNK to β-actin (total JNK/β-actin) are shown in the middle graphs, and the expression levels of β-arrestin2 are shown in the lower graphs in (**a**,**b**). Assays were repeated twice. Values represent mean ± SE of data obtained from four independent protein samples. Individual data are shown as scatter plots. * *p* < 0.05, * *p* < 0.05 vs. control siRNA without histamine treatment; one-way ANOVA and Tukey’s test.

To explore whether H_1_ receptor-mediated JNK phosphorylation might involve arrestin-mediated signal transduction processes, we examined the effects of a GRK2/3 inhibitor (30 µM cmpd101), β-arrestin2 knockdown with siRNA, a clathrin inhibitor (high concentration of sucrose; 0.32 M), and a dynamin inhibitor (100 µM dynasore) on histamine-induced JNK phosphorylation in CHO cells expressing WT and S487TR (Figure 7 and Figure 8). However, these inhibitors did not significantly affect histamine-induced JNK phosphorylation in CHO cells. Hence, histamine-induced JNK phosphorylation was not mediated by the GRK/arrestin/clathrin/dynamin-dependent pathway. These results were consistent with the observation that histamine failed to stimulate JNK phosphorylation in CHO cells expressing arrestin-biased S487A (Figure 3d).

Thus, H_1_ receptor-mediated JNK phosphorylation appears to be regulated by the G_q_ protein/Ca^2+^/PKC-dependent pathway but not by the GRK/arrestin/clathrin/dynamin-dependent pathway.

## 3. Discussion

### 3.1. Mechanisms of H_1_ Receptor-Mediated Phosphorylation of JNK in CHO Cells

In this study, we evaluated the mechanisms underlying histamine-induced JNK phosphorylation in CHO cells expressing WT human H_1_ receptors and their G_q_ protein- and arrestin-biased C-terminal mutants, S487TR and S487A, respectively. We found that histamine-induced JNK phosphorylation is regulated by the G_q_ protein/Ca^2+^/PKC-dependent pathway and by the GRK/arrestin/clathrin/dynamin-independent pathway via the activation of H_1_ receptors. This is in sharp contrast to our previous finding that H_1_ receptor-mediated ERK phosphorylation is regulated by G_q_ protein/Ca^2+^/PKC- and GRK/arrestin/clathrin/Raf/MEK-mediated pathways [43]. The G_q_ protein-dependent pathway via the activation of H_1_ receptors appeared to induce the phosphorylation of both ERK and JNK, whereas the arrestin-dependent pathway appeared to selectively mediate the phosphorylation of ERK rather than JNK. Although the mechanisms responsible for the preferential activation of ERK to JNK by arrestins remain to be clarified, it is possible that the phosphorylation pattern established by GRKs on H_1_ receptors serves as a barcode, which determines subsequent signal transduction pathways via the H_1_ receptor–arrestin complex [11,12,13]. However, the GRK-mediated phosphorylation sites of H_1_ receptors remain to be clarified.

### 3.2. Physiological and Pathophysiological Roles of JNK Phosphorylation

JNK signaling regulates a broad range of physiological processes, including cell proliferation, differentiation, survival, apoptosis, and inflammation [44]. Accordingly, SP600125, a selective inhibitor of JNK, reduces inflammatory cell egress into the airway lumen after a single allergen exposure [45] as well as depression-like behaviors accompanied by increased proinflammatory cytokine expression in rats [46]. Not only ERK-dependent- and JNK-dependent pathways mediate histamine-induced inflammatory reactions via the production of inflammatory cytokines [27,35]. These results suggest that JNK signaling plays a key role in histamine-induced inflammatory responses.

Together with our previous findings that H_1_ receptor-mediated ERK phosphorylation is regulated by G_q_ proteins in the early phase and by arrestins in the late phase [43], it is possible that the early phase of H_1_ receptor-mediated inflammatory responses involves the G_q_ protein-dependent activation of both ERK and JNK. In contrast, the arrestin-dependent activation of ERK with a lack of JNK phosphorylation at the late stage may have different physiological and pathophysiological outcomes from those of G_q_ protein-dependent pathways, as differences in the spatial patterns of G protein- and arrestin-mediated ERK activation may be involved in distinct physiological endpoints [47].

Since MAP kinase pathways are known to regulate various transcriptional factors including NF-κB, STAT3, and CREB, we consider that the roles of H_1_ receptor-mediated ERK- and JNK-signaling pathways in the activation of these transcriptional factors should be clarified in detail. Furthermore, the evaluation of H_1_ receptor-mediated ERK- and JNK-signaling pathways in human cells or tissues endogenously expressing H_1_ receptors is needed to validate our findings using CHO cells expressing G_q_ protein- and arrestin-biased H_1_ receptors.

Nevertheless, to the best of our knowledge, this study provides the first evidence that H_1_ receptor-mediated JNK phosphorylation is regulated by G proteins, but not by arrestins, potentially determining the developmental processes of histamine-induced inflammatory responses. Further investigations may reveal pharmacological and therapeutic aspects based on the biased agonism of H_1_ receptors.

## 4. Materials and Methods

### 4.1. Preparation of CHO Cells Expressing WT and Mutant Human Histamine H_1_ Receptors

The experimental gene-handling protocols were approved by the Institutional Safety Committee for Recombinant DNA Experiments at the Meiji Pharmaceutical University (No. 1209). CHO-K1 cells (RCB0285, RRID: CVCL_0214) were obtained from the RIKEN Bioresource Center (Tsukuba, Ibaraki, Japan), and expression vectors (3xHA hH1R/pcDNA3.1(+)) for WT human histamine H_1_ receptors tagged with three molecules of hemagglutinin (HA: YPYDVPDYA) at the N-terminal were purchased from the Missouri S&T cDNA Resource Center (Rolla, MO, USA). Expression vectors for the C-terminal mutants of WT, S487TR, or S487A, in which the Ser487 residue of WT was truncated or mutated to alanine, respectively, were constructed using the PrimeSTAR mutagenesis basal kit (Takara Bio, Otsu, Shiga, Japan) and Mastercycler Gradient (Eppendorf, Hamburg, Germany), based on 3xHA hH1R/pcDNA3.1(+), according to the manufacturers’ protocols. The nucleotide sequences of the mutated H1 receptor genes were confirmed using the ABI PRISM Genetic Analyzer 310A and the ABI PRISM BigDye Terminator ver. 3.0 (Applied Biosystems, Tokyo, Japan). CHO cells stably expressing WT, S487TR, or S487A were incubated in Dulbecco’s modified Eagle’s medium (Gibco, Gland Island, NY, USA) containing 10% (*v*/*v*) fetal bovine serum (Biowest, Nuaillé, France) in 150 cm^2^ culture flasks (BM Bio, Tokyo, Japan) at 37 °C in CO_2_ incubators (5% CO_2_). Confluent cells were dissociated with trypsin/EDTA (Sigma-Aldrich, St. Louis, MO, USA) and re-seeded in 6-well culture plates (Corning, NY, USA) for subsequent experiments.

### 4.2. Drug Treatments

Drug treatments were administered as in our previous study [42]. Briefly, CHO cells were pre-incubated in a serum-free medium for 48 h before drug treatment. For examining time courses of the histamine-induced phosphorylation of JNK, CHO cells were treated with 100 µM histamine (Sigma-Aldrich) for indicated time periods (15 s–24 h) at 37 °C, and the reaction was terminated by adding ice-cold phosphate-buffered saline (PBS) (Sigma-Aldrich) immediately after the removal of the reaction medium. To investigate the effects of various inhibitors, CHO cells were treated with 100 µM histamine for 30 min in the presence and absence (vehicle) of the inhibitors. Inhibitors used were the following: histamine H_1_ receptor antagonists, ketotifen (Sigma-Aldrich) and diphenhydramine (Sigma-Aldrich) [43]; a G_q_ protein inhibitor, YM-254890 (FUJIFILM Wako Pure Chemical Corporation, Osaka, Japan) [48]; an intracellular Ca^2+^ chelator, BAPTA-AM (Abcam, Cambridge, UK) [49]; a protein kinase C (PKC) inhibitor, GF109203X (Sigma-Aldrich) [50]; a GRK2/3 inhibitor, cmpd101 (Hello Bio, Bristol, UK) [51]; and a dynamin inhibitor, dynasore (Sigma-Aldrich) [52]. These test drugs were dissolved in 10% dimethyl sulfoxide (DMSO) (FUJIFILM Wako Pure Chemical Corporation) and the final concentration of DMSO in the reaction medium was 0.1%. Hypertonic conditions aiming to inhibit the formation of clathrin-coated pits were induced using 0.32 M sucrose (FUJIFILM Wako Pure Chemical Corporation) [53].

### 4.3. Immunoblotting

CHO cells were collected and homogenized in 100 µL radioimmunoprecipitation assay buffer containing protease and phosphatase inhibitor cocktails (Nacalai Tesque, Kyoto, Japan). Cell lysates were centrifuged at 20,000× *g* for 10 min (KUBOTA, Model 3700, Tokyo, Japan), and supernatants were collected as protein samples for immunoblotting analyses. Protein content was determined using a Pierce bicinchoninic acid protein assay kit (Thermo Fisher Scientific, Waltham, MA, USA). Protein samples of 10 μg were applied to each lane and were electrophoresed on a 7.5% polyacrylamide gel and transferred onto polyvinylidene difluoride membranes (Millipore, Burlington, MA, USA). The proteins transferred to the membranes were reacted with primary antibodies for JNK (Cell Signaling Technology, Danvers, MA, USA), phosphorylated JNK (Cell Signaling Technology), and β-arrestin2 (Santa Cruz Biotechnology Inc., Dallas, TX, USA) and then reacted with peroxidase-conjugated secondary antibody (Millipore) for detection with a chemiluminescence kit (Chemi-Lumi One^®®^ L, Nacalai Tesque). The intensity of the protein bands was determined using ImageJ software (version 1.53c, https://imagej.nih.gov/ij/, Accessed on 26 June 2020). Membranes were also reacted with the primary antibody for β-actin (Abcam) and peroxidase-conjugated secondary antibody (Santa Cruz Biotechnology Inc.) as an internal standard. The molecular weights of proteins were evaluated using Precision Plus Protein™ Kaleidoscope™ Prestained Protein Standards (Bio-Rad, Hercules, CA, USA).

### 4.4. β-Arrestin2 Knockdown

CHO cells were treated with control siRNAs or β-arrestin2 siRNAs in an siRNA transfection medium containing siRNA transfection reagent for 48 h according to the supplier’s protocol (Santa Cruz Biotechnology Inc.).

### 4.5. Immunocytochemistry

CHO cells that grew on glass coverslips in 12-well plates (Corning Costar Corp., Cambridge, MA, USA) were fixed with PBS containing 4% paraformaldehyde and permeabilized with PBS containing 0.1% Triton-X-100. To detect WT and mutant H_1_ receptors, CHO cells were labeled with primary antibodies against HA (16B12; BioLegend, Tokyo, Japan) and Alexa-Fluor-568-conjugated secondary antibodies (Thermo Fisher Scientific, Waltham, MA, USA). To detect nuclei, CHO cells were labeled with 4′,6-diamidino-2-phenylindole (DAPI) (Wako Pure Chemical Industries, Osaka, Japan). Confocal immunofluorescence microscopy was performed using a laser scanning microscope (AX; Nikon, Tokyo, Japan).

### 4.6. Statistical Analysis

The results are presented as mean ± standard error (SE). All data were obtained from four independent experiments. Normality tests were performed with normal probability plots by using Ekuseru-Toukei (BellCurve for Excel, Social Survey Research Information Co., Ltd., Tokyo, Japan), which indicated that all data were within a normal distribution. Statistical significance was determined by one-way analysis of variance (ANOVA), followed by a post hoc test using Ekuseru-Toukei software. Results were considered significant at *p* < 0.05.

## 5. Conclusions

In this study, it was revealed that histamine-induced and H_1_ receptor-mediated JNK phosphorylation was regulated by G_q_ protein/Ca^2+^/PKC-dependent but GRK/arrestin/clathrin-independent pathways in CHO cells. Further investigations may provide novel insights into how developmental processes of allergic and inflammatory responses are regulated by the H_1_ receptor-mediated differential activation of MAP kinases via G_q_ proteins and arrestins.

## Data Availability

The datasets used and/or analyzed during the current study are included in this article and available from the corresponding author upon reasonable request.

## Supplementary Materials

The following supporting information can be downloaded at: https://www.mdpi.com/article/10.3390/ijms25063395/s1.

## Abbreviations

CHO	Chinese hamster ovary
ERK	Extracellular signal-regulated kinase
GPCR	G protein-coupled receptor
GRK	G protein-coupled receptor kinase
JNK	c-Jun N-terminal kinase
MAP	Mitogen-activated protein
PBS	Phosphate-buffered saline
