# Supplementary material for: Histamine H1 Receptor-Mediated JNK Phosphorylation Is Regulated by Gq Protein-Dependent but Arrestin-Independent Pathways
Source: Int J Mol Sci. 2024 Mar 17;25(6):3395. doi: 10.3390/ijms25063395 (PMC10970263; doi:10.3390/ijms25063395)
Supplement: Supplementary file 1 [file ijms-25-03395-s001.zip › Supplemental Figure S1.pdf]

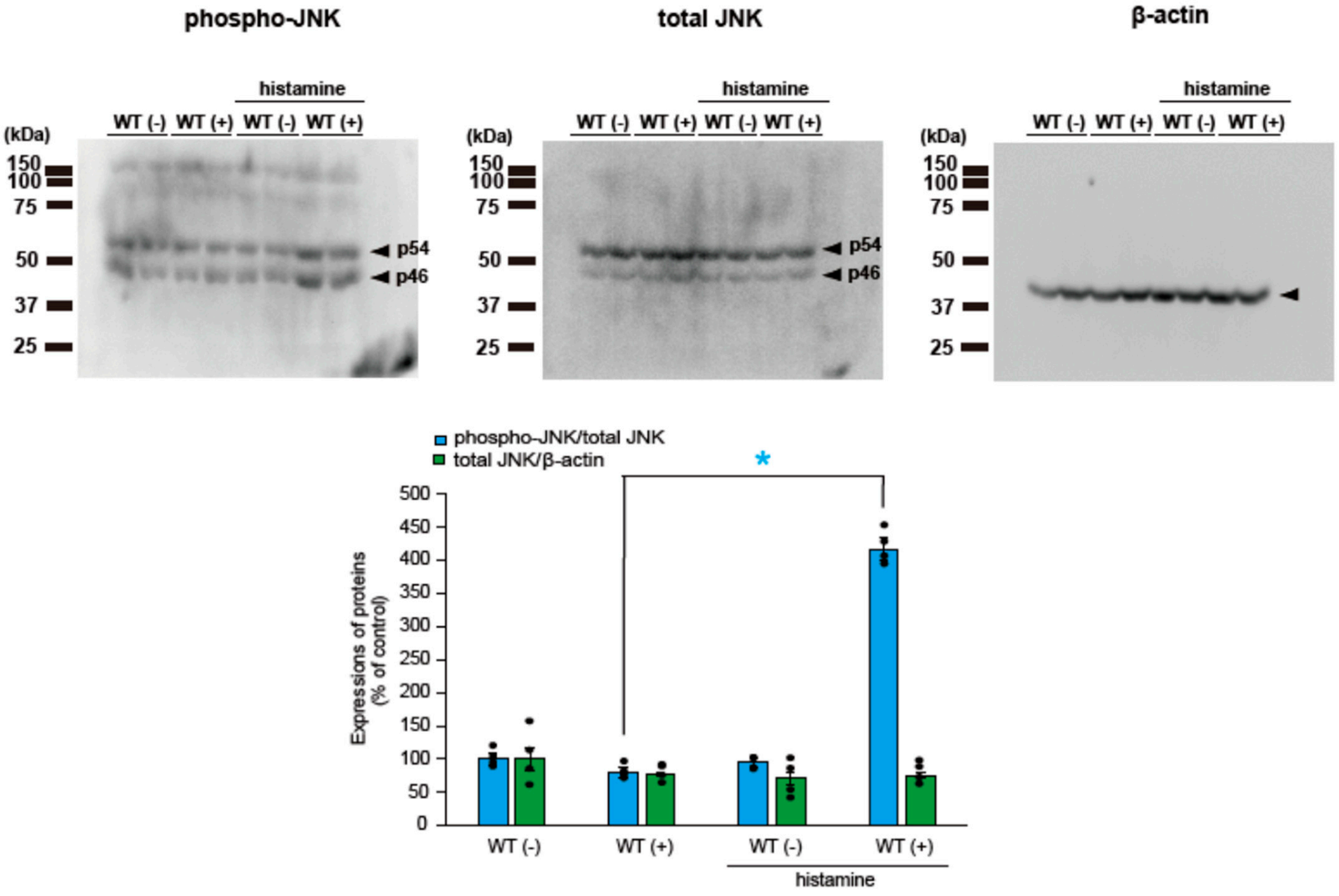

Supplemental Figure S1. Comparison of histamine-induced JNK phosphorylation in CHO cells non-expressing (WT (-)) and expressing WT H_1 receptors (WT (+)). WT (-) and WT (+) CHO cells were stimulated with or without 100 μM histamine for 30 min and then protein extracts from the cells were subjected to immunoblot analyses. Typical untruncated immunoblot images of phosphorylated JNK (phospho-JNK, p46 and p54) (left), total JNK (p46 and p54) (middle), and β-actin (40 kDa) (right) are shown in the upper panels with arrowheads. The molecular weights are shown in the left side of images. Histamine-induced changes in the ratios of phosphorylated JNK to total JNK (phospho-JNK/total JNK) and total JNK to β-actin (total JNK/β-actin) are shown as percentages of the control in the lower graphs. Assays were repeated twice. Values represent mean ± SE of data obtained from four independent protein samples. Individual data are shown as scatter plots. *$P < 0.05$ vs. WT (+) without histamine treatment; one-way ANOVA and Tukey's test.
