# Supplementary material for: Histamine H1 Receptor-Mediated JNK Phosphorylation Is Regulated by Gq Protein-Dependent but Arrestin-Independent Pathways
Source: Int J Mol Sci. 2024 Mar 17;25(6):3395. doi: 10.3390/ijms25063395 (PMC10970263; doi:10.3390/ijms25063395)
Supplement: Supplementary file 1 [file ijms-25-03395-s001.zip › Supplemental Figure S2.pdf]

(a) WT

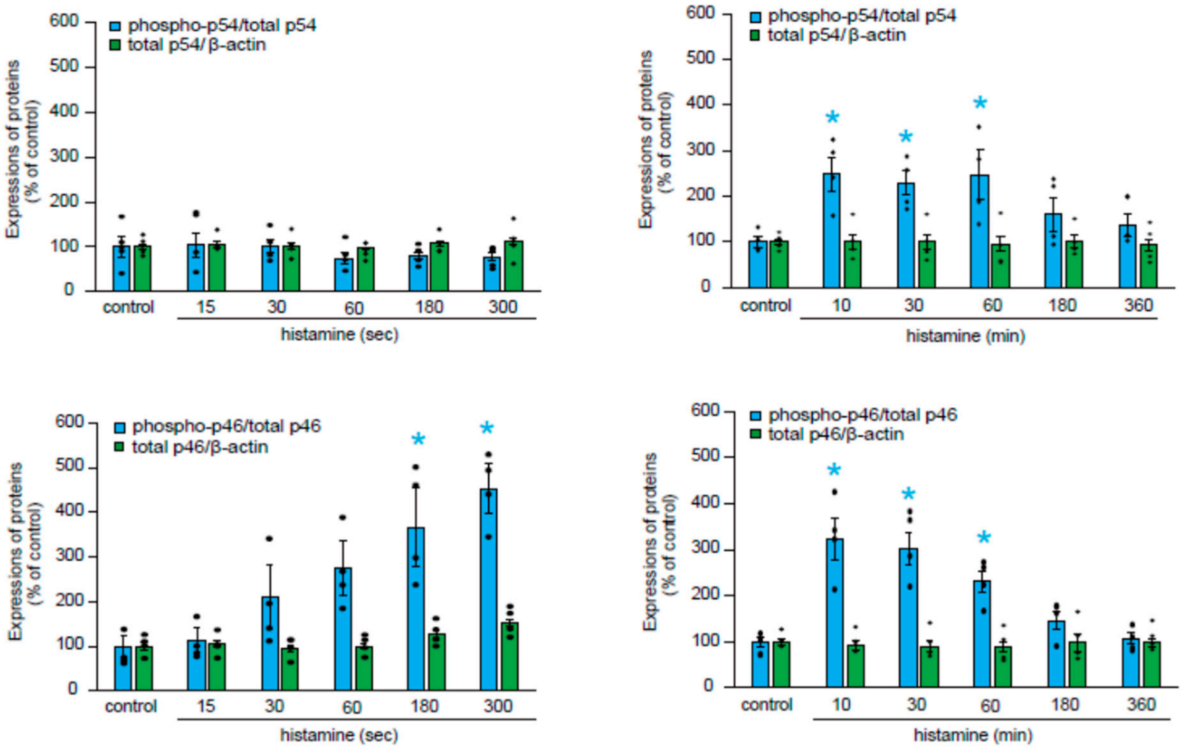

phospho-p54/total p54
total p54/ β-actin
Expressions of proteins (% of control)
control
15
30
60
180
300
histamine (sec)
10
30
60
180
360
histamine (min)
phospho-p46/total p46
total p46/β-actin

(b) S487TR

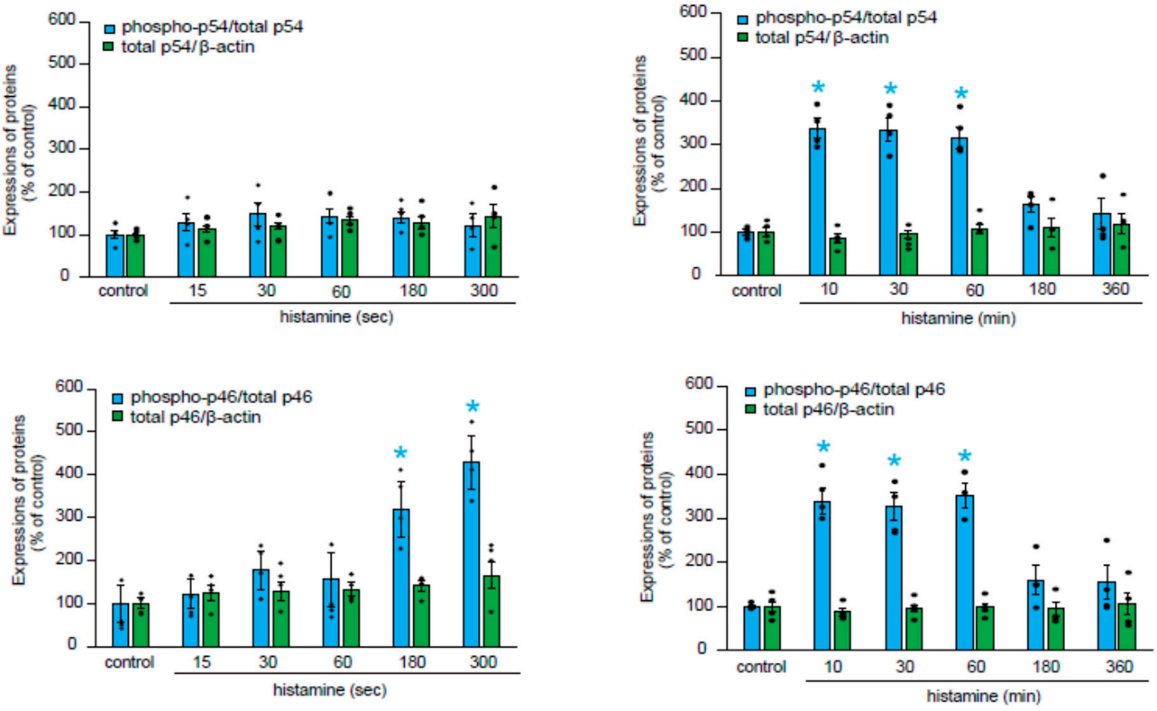

phospho-p54/total p54
total p54/ β-actin
Expressions of proteins (% of control)
control
15
30
60
180
300
histamine (sec)
10
30
60
180
360
histamine (min)
phospho-p46/total p46
total p46/β-actin

Supplemental Figure S2. Time courses of histamine-induced p46 and p54 phosphorylation. CHO cells expressing WT (a) and S487TR (b) were stimulated with or without (control) 100 μM histamine for 15–300 sec (left) and 10–360 min (right), and then protein extracts from the cells were subjected to immunoblot analyses. Histamine-induced changes in the ratios of phosphorylated p54 to total p54 (phospho-p54/total p54) and total p54 to β-actin (total p54/β-actin) are shown as percentages of the control without histamine treatment in the upper graphs in (a) and (b). Histamine-induced changes in the ratios of phosphorylated p46 to total p46 (phospho-p46/total p46) and total p46 to β-actin (total p46/β-actin) are shown as percentages of the control without histamine treatment in the lower graphs in (a) and (b). Assays were repeated four times. Values represent mean ± SE of data obtained from four independent protein samples. Individual data are shown as scatter plots. *$P < 0.05$ vs. control; one-way ANOVA and Dunnett's test.
