# Supplementary material for: Histamine H1 Receptor-Mediated JNK Phosphorylation Is Regulated by Gq Protein-Dependent but Arrestin-Independent Pathways
Source: Int J Mol Sci. 2024 Mar 17;25(6):3395. doi: 10.3390/ijms25063395 (PMC10970263; doi:10.3390/ijms25063395)
Supplement: Supplementary file 1 [file ijms-25-03395-s001.zip › Supplemental Figure S3.pdf]

(a) WT

Histamine 15-300 sec (n=4)

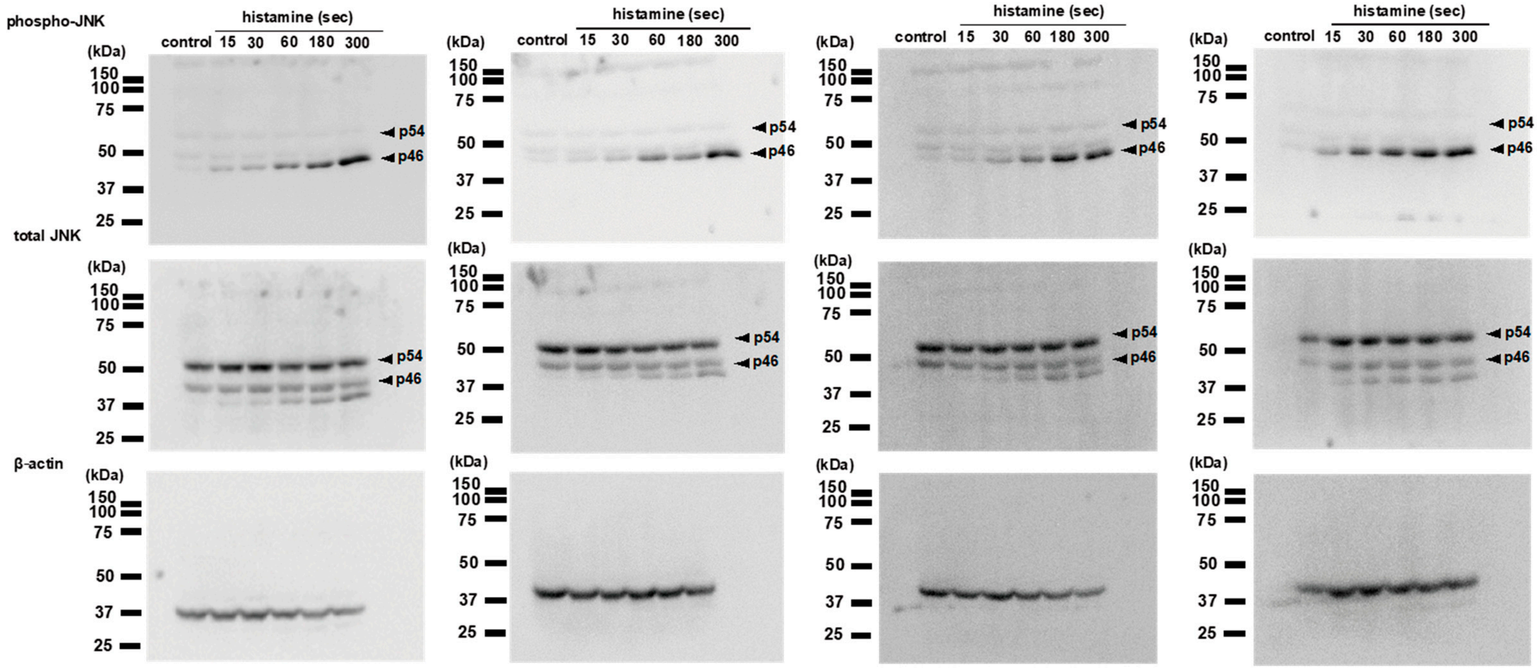

Histamine 10-360 min (n=4)

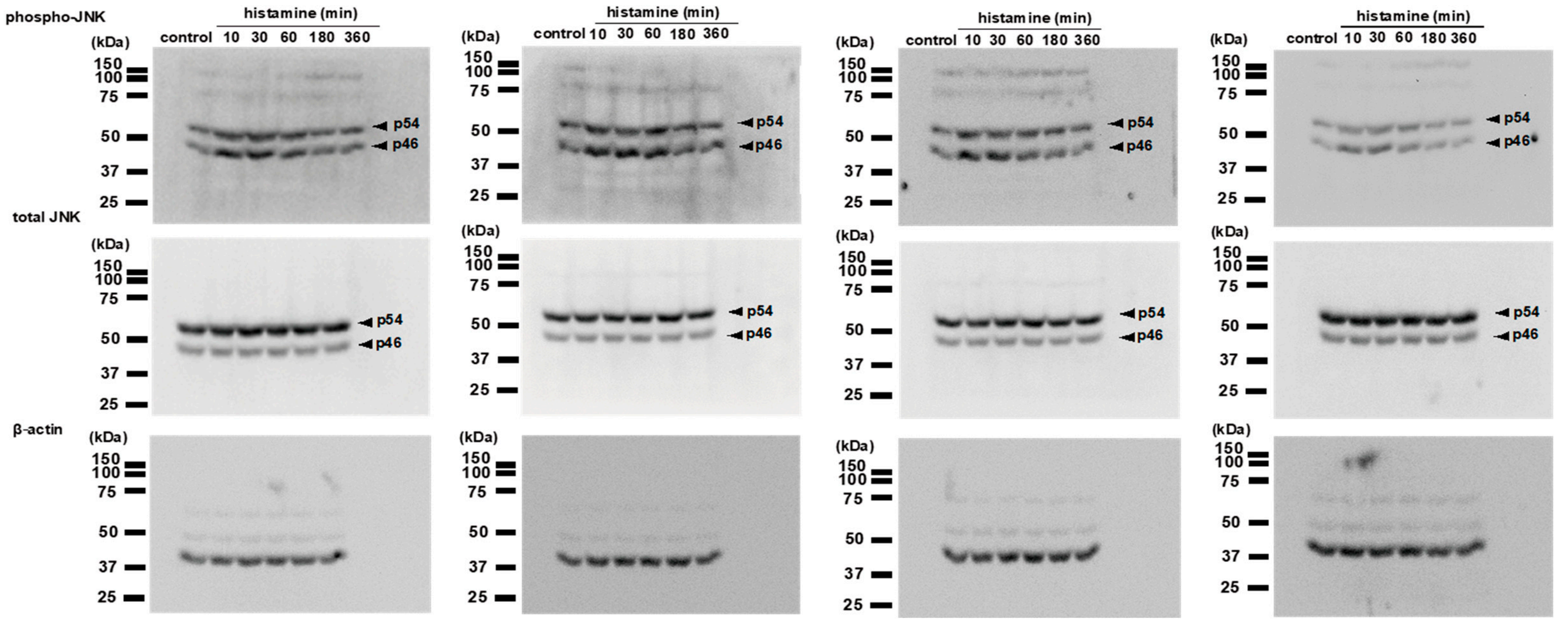

Histamine 12 and 24 h (n=4)

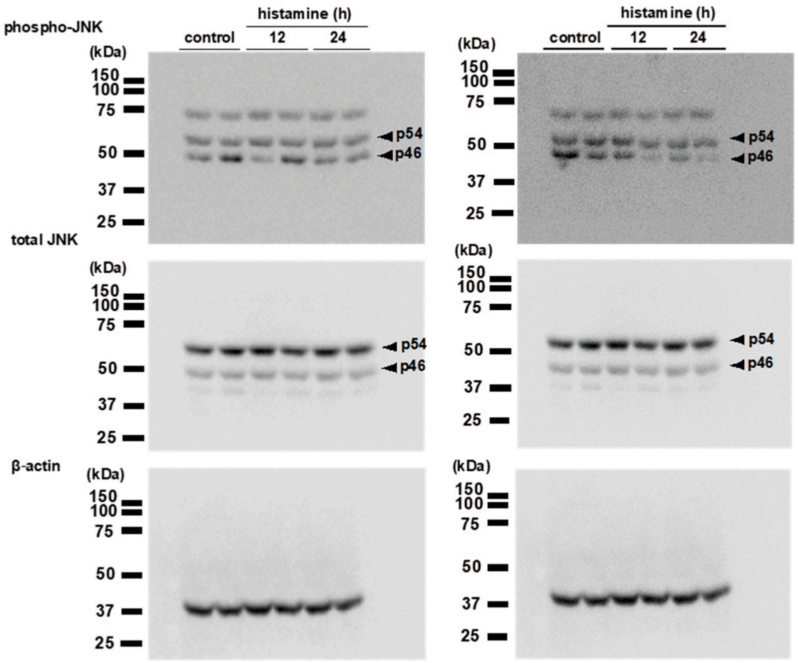

(b) S487TR

Histamine 15-300 sec (n=4)

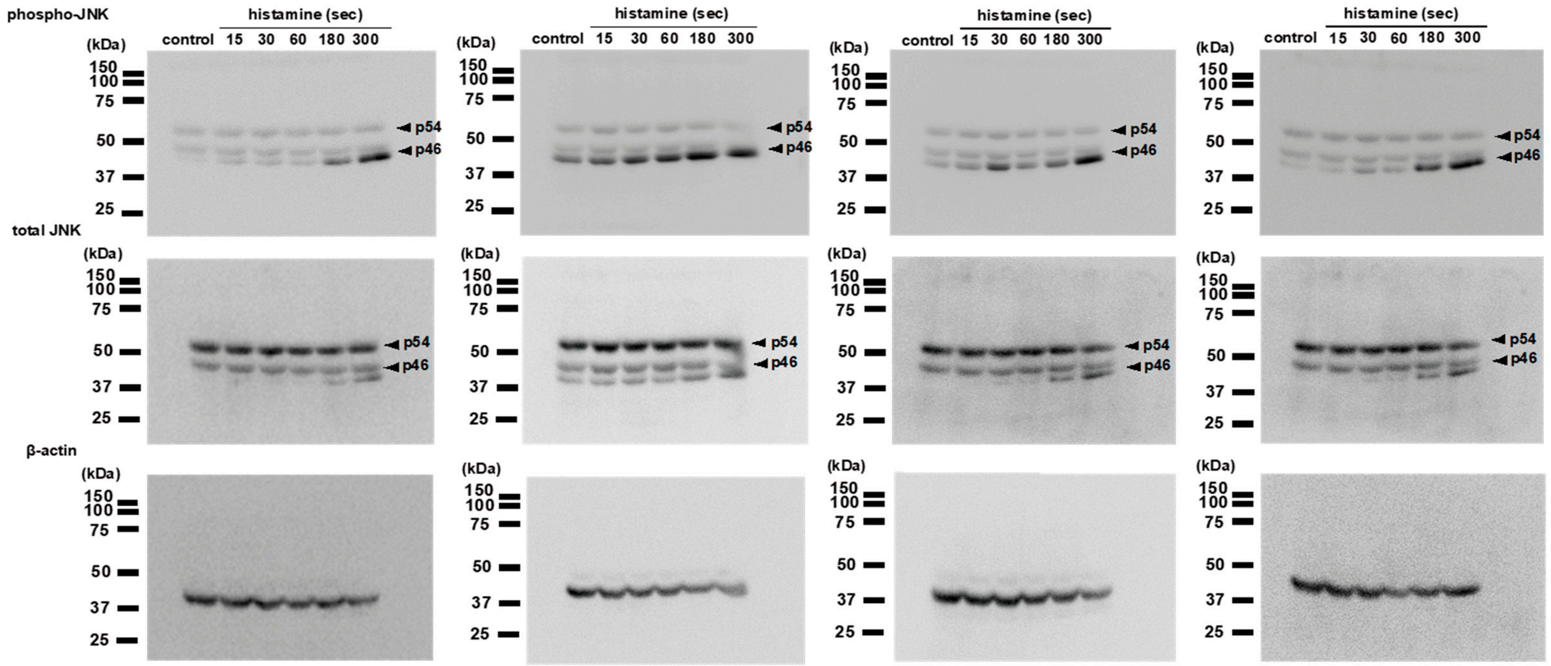

Histamine 10-360 min (n=4)

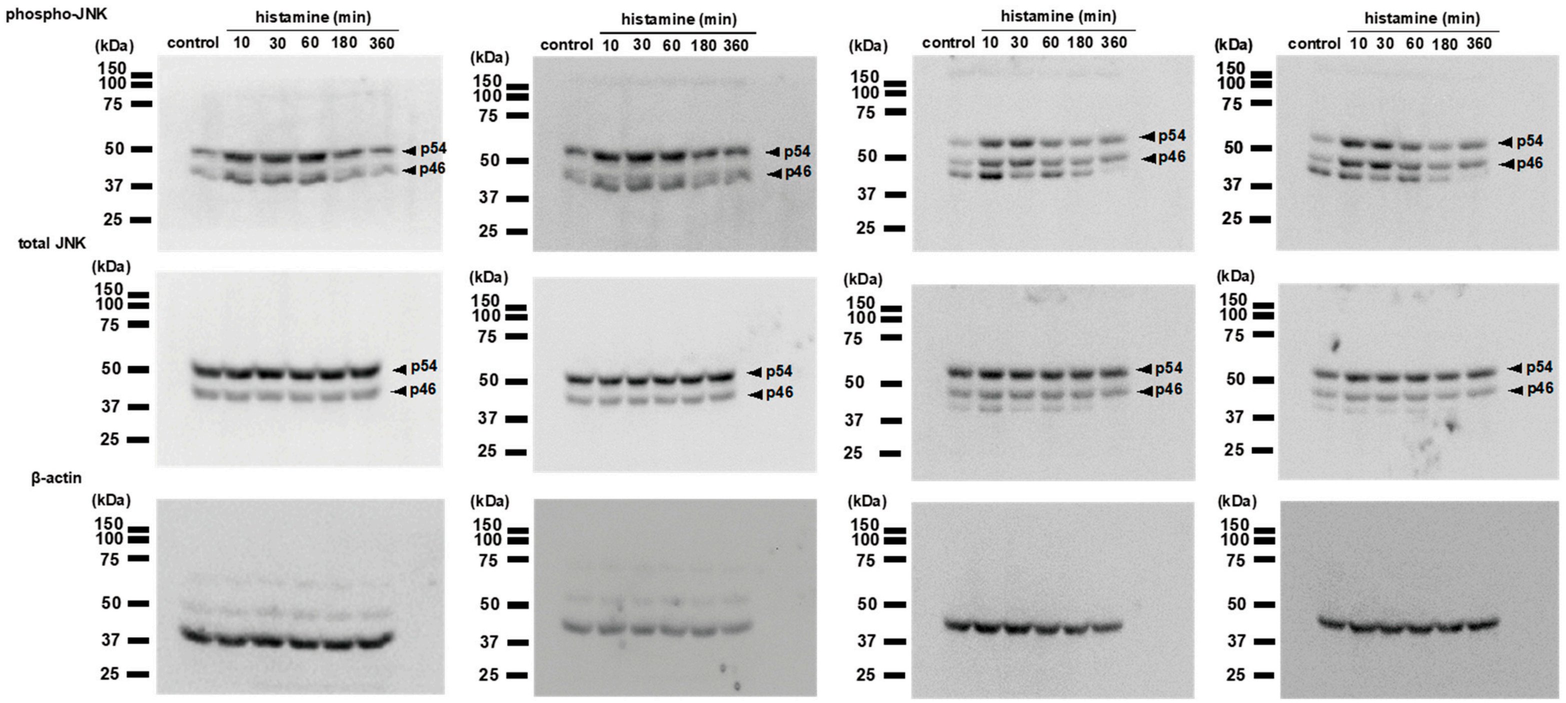

Histamine 12 and 24 h (n=4)

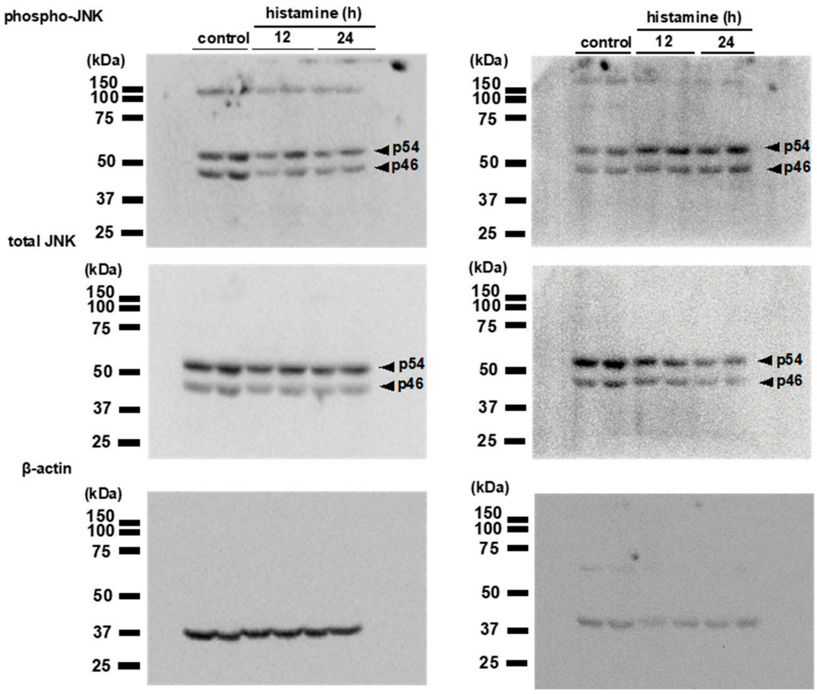

(c) S487A

Histamine 15-300 sec (n=4)

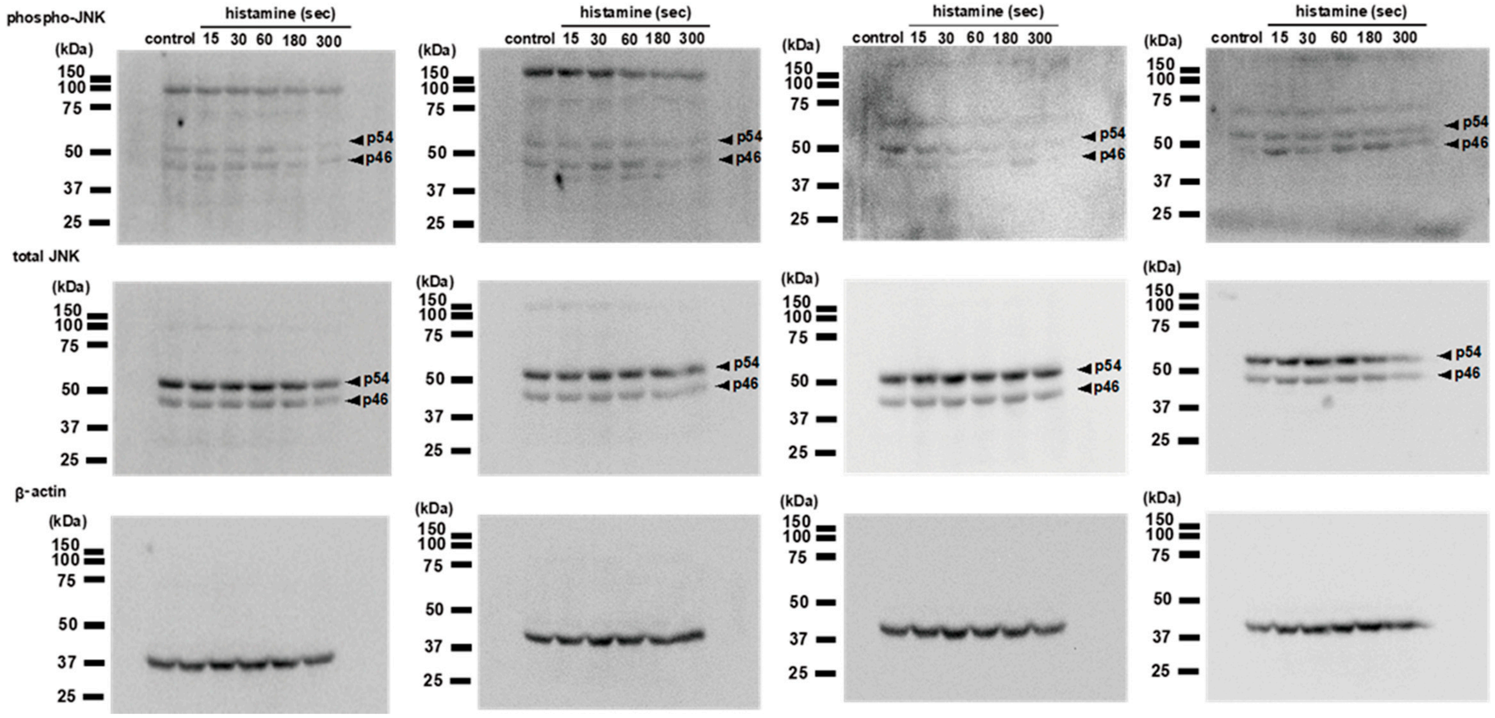

Histamine 10-360 min (n=4)

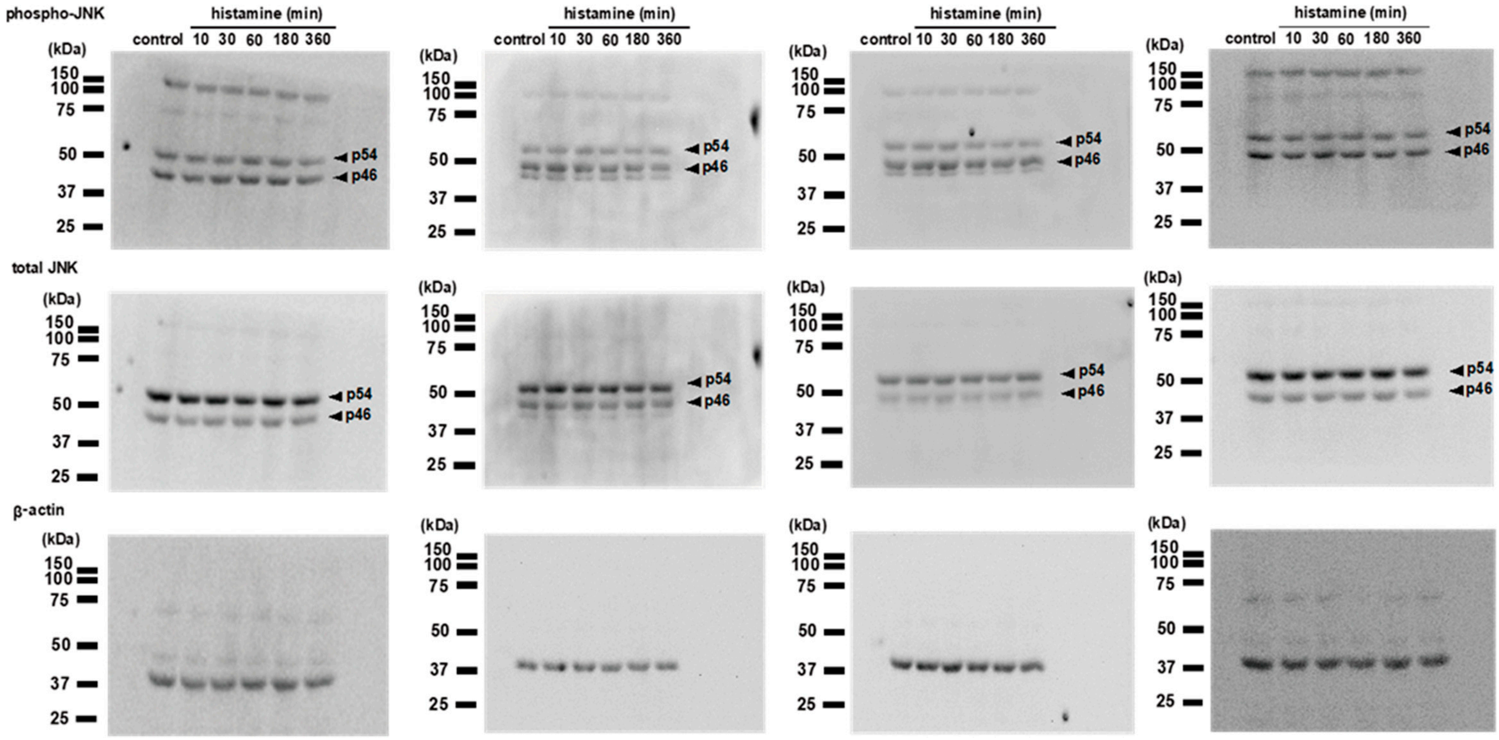

Histamine 12 and 24 h (n=4)

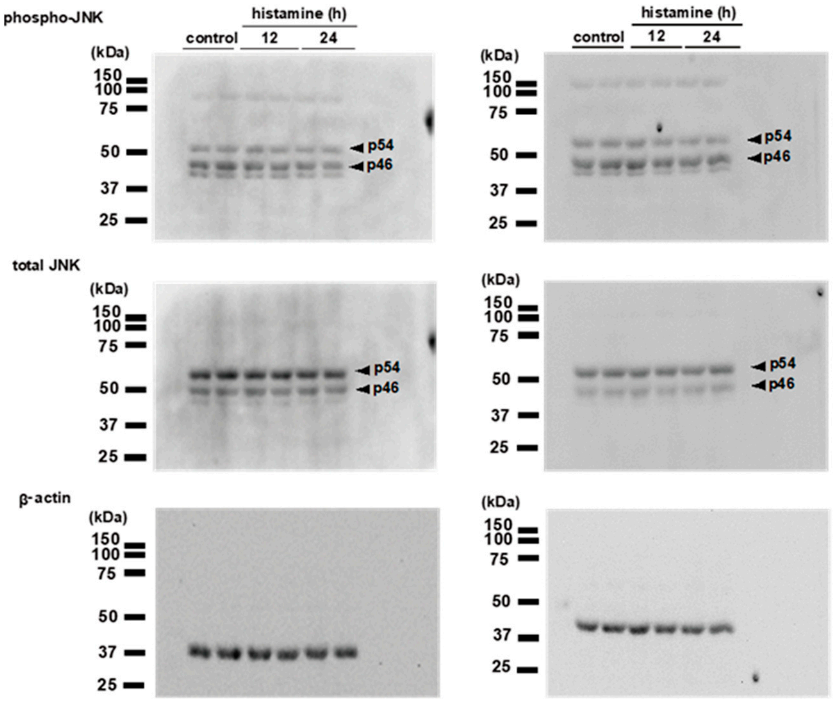

Supplemental Figure S3. Uncropped immunoblot images obtained from experiments to evaluate time courses of histamine-induced JNK phosphorylation. CHO cells expressing WT (a), S487TR (b), and S487A (c) were stimulated with or without (control) 100 μM histamine for 15–300 sec , 10–360 min, and 12–24 h and then protein extracts from the cells were subjected to immunoblot analyses. All of uncropped immunoblot images (n=4) of phosphorylated JNK (phospho-JNK), total JNK, and β-actin are shown in (a)–(c).
